# Atypical Foreign Body Ingestion Leading to Evidence of Gastroparesis

**DOI:** 10.7759/cureus.91739

**Published:** 2025-09-06

**Authors:** Ryan Burd, Ryan Gilbertson, Sandeep Gill, John Michael Vincent Coralde, Indraneel Chakrabarty

**Affiliations:** 1 Internal Medicine, Southwest Healthcare Medical Education Consortium, Temecula, USA; 2 Gastroenterology, Southwest Healthcare Medical Education Consortium, Temecula, USA

**Keywords:** digestion, endoscopy, esophagogastroduodenoscopy (egd), foreign body, gastroparesis

## Abstract

Foreign body (FB) ingestion in adults commonly occurs in the context of dining, substance use, and psychiatric disorders. Gastroparesis is defined as the delayed emptying of gastric contents without mechanical obstruction. This case report presents a 38-year-old male who unintentionally ingested the tip of a metal knife, with subsequent imaging supporting evidence of gastroparesis. Previously undiagnosed gastroparesis is suspected to have spared this patient from life-threatening consequences of FB ingestion, including gastrointestinal perforation and hemorrhage. This report underscores the value of timely multidisciplinary coordination, imaging, and intervention while raising awareness of atypical presentations of gastroparesis.

## Introduction

The intentional or unintentional swallowing of foreign bodies (FBs) affects approximately 120,000 people globally and is commonly seen in the context of dining, digestive disease, alcohol and drug use, and psychiatric disorders in adults [1-3]. While many FBs pass through the gastrointestinal tract spontaneously, 10-20% require endoscopic intervention, while less than 1% require surgical intervention [4]. Gastroparesis is defined as delayed gastric emptying in the absence of mechanical obstruction, often resulting in early satiety, postprandial fullness, nausea, vomiting, and bloating [5]. While the most common etiology of gastroparesis is diabetes mellitus, accounting for approximately one-third of cases [6], studies estimate up to 1.8% of the population to be affected, with only 0.2% being diagnosed [7-9]. In the following case report, we present a 38-year-old male with a distant history of intermittent early satiety, who ingested a FB in the form of a metallic knife tip unknowingly while eating. The patient’s clinical presentation, associated imaging findings, and absence of mechanical obstruction observed during esophagogastroduodenoscopy (EGD), along with retention of gastric contents for over six hours, provided evidence of undiagnosed idiopathic gastroparesis. Gastroparesis may have inadvertently prevented the progression of this FB from passing through the pylorus and becoming unretrievable, avoiding emergent complications of gastrointestinal perforation, laceration, and hemorrhage. Furthermore, this report highlights the challenges associated with retrieving an atypical FB, which, when superimposed with gastroparesis, was managed successfully with complete avoidance of complications.

## Case presentation

A 38-year-old male with a past medical history significant for intravenous (IV) methamphetamine use, last used 10 years prior to admission, and a surgical history of appendectomy 20 years prior to admission, presented to the emergency department of a community hospital approximately three hours after unintentionally ingesting the tip of a knife. The patient reported that he consumes frozen protein nutritional bars, typically cut into smaller pieces for ease of consumption. While cutting, the patient unknowingly broke off the tip of a knife into a piece of a bar, ingesting it despite noticing an unusual sensation upon mastication. The patient was unable to locate the source of this sensation after oral examination and later noticed the missing tip of the knife, prompting presentation to the emergency department for evaluation. The patient denied any use of medicinal or herbal supplements, vitamins, or hormonal therapies, and did not take any scheduled medications at the time of presentation. The patient denied any additional previously diagnosed medical conditions, including endocrine or neurologic disorders. Furthermore, the patient denied any remarkable family history of medical diagnoses. 

Upon review of systems and initial evaluation, the patient admitted to intermittent early satiety and post-prandial bloating with large meals occurring for multiple years, for which he never sought medical evaluation. The patient denied oropharyngeal, throat, chest or abdominal pain, fever, chills, nausea, vomiting, indigestion, hematemesis, hematochezia, or melena.

**Figure 1:**
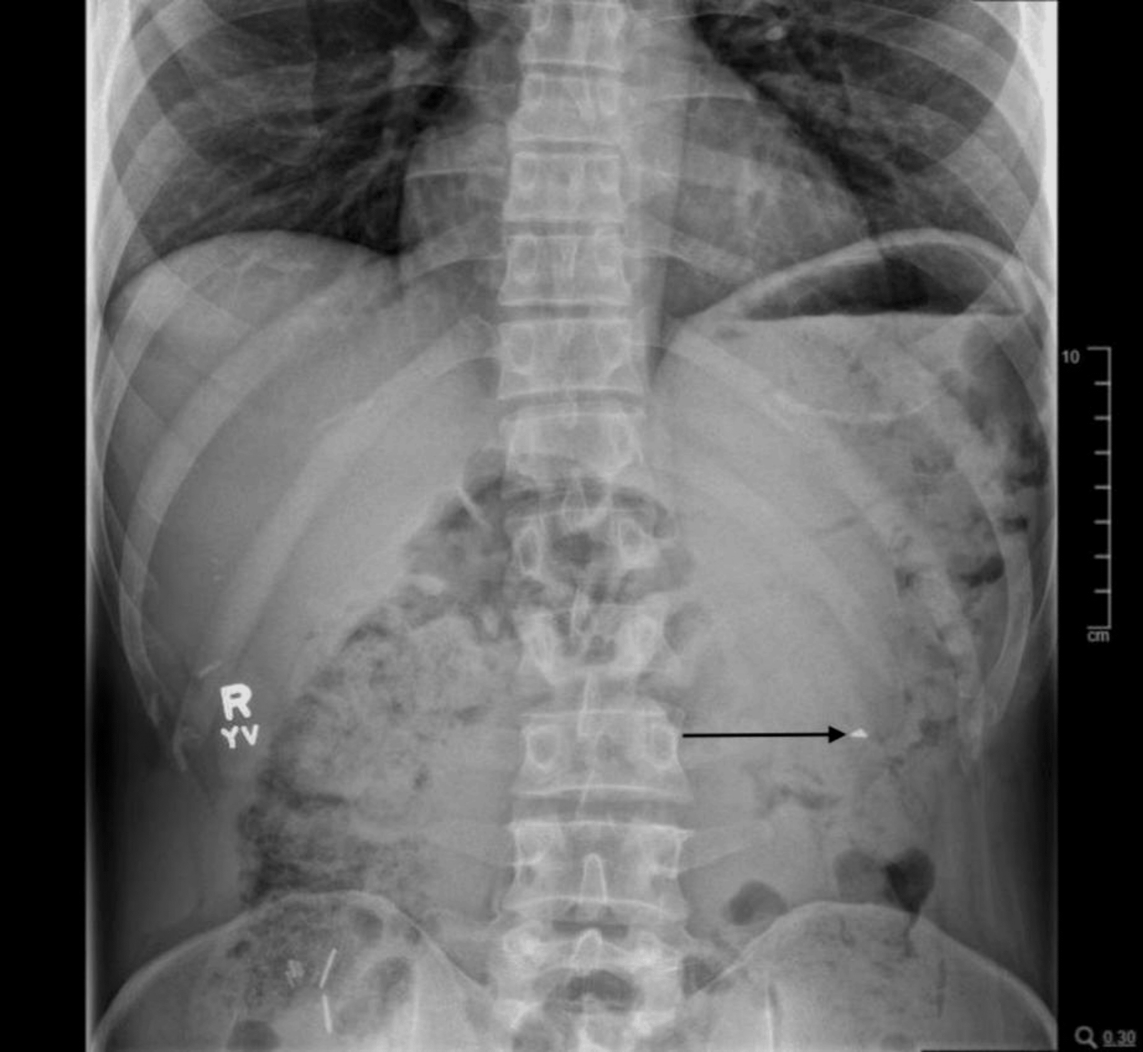
Abdominal radiograph of FB ingestion (see arrow) Abdominal radiograph showing FB in the distal gastric body. FB, foreign body

**Figure 2:**
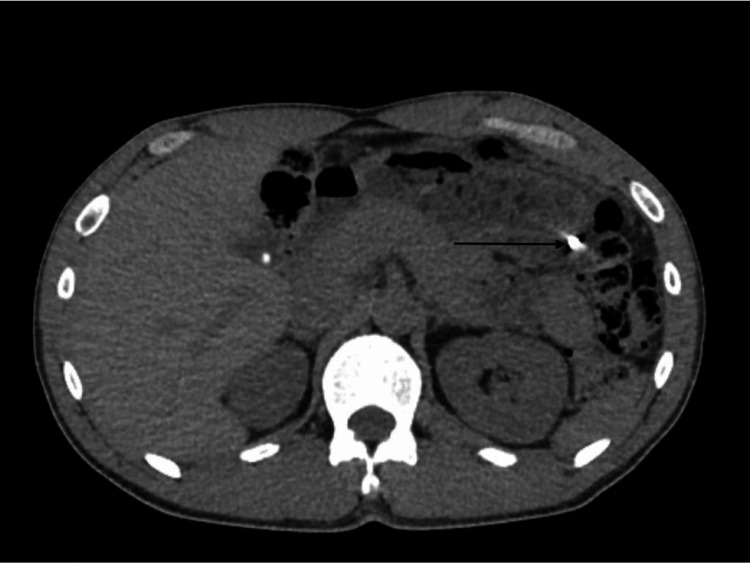
CT scan of FB ingestion (see arrow) Axial abdominal CT scan showing metallic artifact from FB in the distal gastric body. FB, foreign body; CT, computed tomography

The patient’s vitals upon presentation were afebrile and hemodynamically stable with a blood pressure of 110/83 mmHg and oxygen saturation of 96% on room air. Complete blood count demonstrated no leukocytosis, anemia, or thrombocytopenia. A complete metabolic panel demonstrated electrolytes within normal limits and no evidence of renal failure, hyperglycemia, or transaminitis. Thyroid-stimulating hormone was within normal limits. Imaging studies in the emergency department included an abdominal radiograph (Figure 1) showing a radiolucent, angular FB in the stomach, the position and shape of which was corroborated on a subsequent computed tomography (CT) scan of the abdomen (Figure 2).

**Figure 3:**
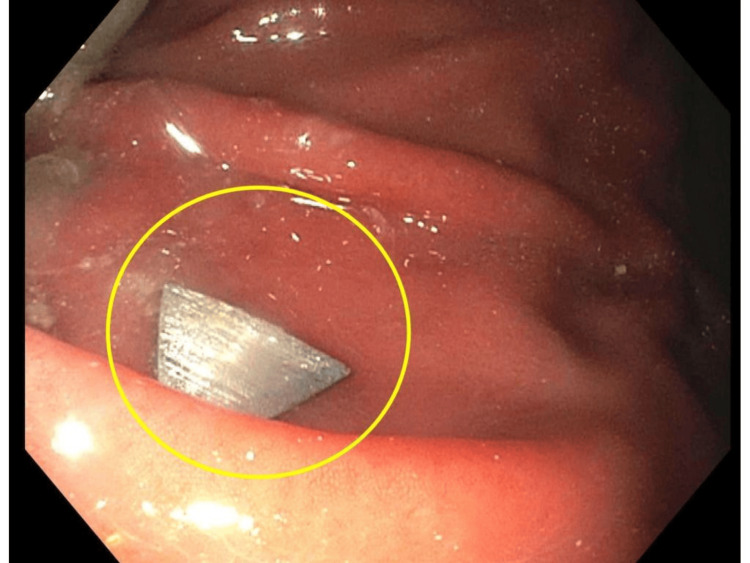
Endoscopic view of FB ingestion (see circle) Unobstructed view of FB within the gastric body. FB, foreign body

The on-call gastroenterology service was subsequently consulted from the emergency department for assistance with management and retrieval of the ingested FB. More than six hours after initial ingestion, the patient was prepared for EGD, which localized the FB within the stomach and proximal to the pyloric sphincter (Figure 3). Endoscopic retrieval of the FB was safely performed using forceps. The patient tolerated this procedure without complications and was safely discharged from the hospital the same day with advised follow-up with a gastroenterologist for outpatient gastric emptying scintigraphy. During outpatient follow-up, the patient underwent gastric emptying scintigraphy, confirming his diagnosis of gastroparesis. He was advised to consume small, frequent meals and was prescribed scheduled metoclopromide to be taken before meals. Symptomatic early satiety and post-prandial bloating reportedly improved.

## Discussion

According to a retrospective review by Wang et al. (2021) of over 1,300 patients diagnosed with FB ingestion, the most commonly reported complication was hemorrhage in 67.8% of patients, followed by perforation in 27.2% [10]. Principal risk factors associated with complications included age greater than or equal to 60, presence of a FB for greater than or equal to 24 hours, and esophageal location of the FB, with odds ratios of 1.54, 2.67, and 2.07, respectively [10]. Among FB ingestions globally, the most commonly ingested objects include fish bones, shells, and other indigestible objects swallowed in the context of eating [11-13].

Successful management of FB ingestion requires appropriate intervention based on object characteristics and location. This case report highlights the retrieval of a metallic knife tip confined to the stomach without progression over six hours after ingestion. Diagnosis of gastroparesis in the absence of traditional risk factors requires the elimination of an initially broad differential diagnostic list, including mechanical obstruction, peptic ulcer disease, malignancy, extrinsic compression, and other diagnoses. Systemic or functional impairments of gastric motility, including hypothyroidism, neurologic, and iatrogenic causes, must also be eliminated, as was done for this patient. The patient's only symptoms suggestive of gastroparesis prior to hospitalization included intermittent early satiety and postprandial bloating. Ultimately, this suspected diagnosis was confirmed on outpatient gastric emptying scintigraphy. While the diagnostic gold standard of gastroparesis is via gastric emptying scintigraphy [14], this study does not require hospitalization or prolonged monitoring, making it ideal for the outpatient setting [15].

Finally, it is thought that gastroparesis played a protective role in the ingestion of this FB, preventing distal migration of the sharp object, peristalsis of which could have led to life-threatening complications. Timely clinical evaluation, imaging, multidisciplinary coordination, and endoscopic strategy are essential to managing such cases effectively.

## Conclusions

This case report illustrates an atypical presentation of accidental FB ingestion with mitigated complications due to previously undiagnosed gastroparesis. Had the object migrated distally, it might have led to perforation or required surgical intervention. The severity of the ingestion in this case report further emphasizes the value of a thorough history and appropriate imaging. While gastroparesis is often associated with discomfort and nutritional concerns, it likely prevented a more dangerous clinical course and serves as a reminder that nuanced clinical presentations demand careful attention, for an underlying disease process may work in the patient’s favor.
